# Coordinate responses to alkaline pH stress in budding yeast

**DOI:** 10.15698/mic2015.06.205

**Published:** 2015-05-22

**Authors:** Albert Serra-Cardona, David Canadell, Joaquín Ariño

**Affiliations:** 1 Departament de Bioquímica i Biologia Molecular & Institut de Biotecnologia i Biomedicina, Universitat Autònoma de Barcelona, Bellaterra 08193, Barcelona, Spain.

**Keywords:** calcineurin, Snf1, PKA, transcriptomics, S. cerevisiae

## Abstract

Alkalinization of the medium represents a stress condition for the budding yeast *Saccharomyces cerevisiae *to which this organism responds with profound remodeling of gene expression. This is the result of the modulation of a substantial number of signaling pathways whose participation in the alkaline response has been elucidated within the last ten years. These regulatory inputs involve not only the conserved Rim101/PacC pathway, but also the calcium-activated phosphatase calcineurin, the Wsc1-Pkc1-Slt2 MAP kinase, the Snf1 and PKA kinases and oxidative stress-response pathways. The uptake of many nutrients is perturbed by alkalinization of the environment and, consequently, an impact on phosphate, iron/copper and glucose homeostatic mechanisms can also be observed. The analysis of available data highlights cases in which diverse signaling pathways are integrated in the gene promoter to shape the appropriate response pattern. Thus, the expression of different genes sharing the same signaling network can be coordinated, allowing functional coupling of their gene products.

## INTRODUCTION

The ability of living cells for adaptation to changes in the environment is crucial for survival. Extracellular pH is a key parameter that influences many aspects of the biology of cells. The budding yeast *Saccharomyces cerevisiae *grows under a relatively broad range of external pH, but proliferates far better at acidic than at neutral or alkaline pH. The maintenance of a suitable acidic environment is achieved by active proton extrusion mediated by the plasma-membrane H^+^-ATPase, encoded by the essential *PMA1* gene 1. In collaboration with the vacuolar H^+^-ATPase, Pma1 is also crucial for maintenance of cytosolic pH homeostasis 2. The electrochemical gradient of protons across the plasma membrane is required for all secondary symporters and antiporters critical for the uptake of different nutrients, including diverse cations, mainly potassium 3,4. In addition, external alkaline pH prompts differentiation programs, such as haploid invasive growth and sporulation, which are both inhibited under acidic growth 5,6. Most *S. cerevisiae* laboratory strains can hardly grow in a medium buffered above pH 8.0-8.2, and alkalinization of the medium has widespread effects on the yeast physiology, as inferred from the very diverse nature of the near 300 mutants known to display growth defects at neutral or moderately alkaline pH 7,8,9. Many of these mutants show vacuolar defects, particularly those lacking structural components of the vacuolar ATPase or those required for its assembly, which are extremely sensitive to even moderate alkalinization of the medium 8,10. In addition, it has been shown that under some circumstances V-ATPase complexes are stabilized when the extracellular pH increases, becoming less susceptible to dissociation, and increasing their activity 11,12. All these evidence clearly indicate that a functional vacuole is required for normal alkaline pH tolerance. Although a vacuolar role in maintaining proper cytosolic pH has been proposed, the necessity for a functional vacuole is not completely understood 13, and has probably a multifactorial nature that will be discussed further in other sections of this review.

The remodeling of gene expression is at the basis of all stress responses in *S. cerevisiae* 14. The influence of alkaline pH on gene expression was first documented in *Aspergillus nidulans*, and later in *Candida albicans*, and the mechanisms behind this response have been characterized in some detail in these organisms 15,16,17. Comparatively, knowledge on the adaptive responses of *S. cerevisiae *to alkaline pH was almost inexistent until early in the last decade 9. However, a wealth of information has been generated since then, allowing the dissection of diverse pathways that are transiently modulated when budding yeast is exposed to environmental alkalinization, which - as expected - entails extensive regulation at the level of gene expression. In this work we describe these pathways and how they interact to shape a transient response that allows adaptation and, eventually, survival of the yeast cell. The knowledge of the adaptive mechanisms to alkaline pH will be useful, not only from the point of view of fundamental research, helping to dissect signaling pathways and their downstream targets, but also for industrial biotechnology, guiding the design of high-pH tolerant strains able to survive in demanding fermentation conditions. In addition, the signals that trigger the morphological change from the *Candida albicans *non-pathogenic yeast form to the pathogenic hyphal form are produced in response to a shift to neutral/alkaline pH. Thus, these pathways are also related to fungal virulence.

### THE CALCINEURIN PATHWAY AND THE ALKALINE pH STRESS RESPONSE

Exposure of yeast cells to alkaline stress promotes a very fast entry of calcium from the environment that requires the Mid1/Cch1 high-affinity calcium transport system 18. Although the precise mechanism is not known, it has been proposed that Mid1 is an stretch-activated cation channel 19,20, whereas Cch1 would be a voltage-gated calcium channel 20,21. Interestingly, alkaline pH activates the Slt2 MAP kinase pathway through the Wsc1 surface sensor 22 (see below, section "OTHER STRESS SIGNALING PATHWAYS ACTIVATED BY ALKALINE pH STRESS"), which subsequently was demonstrated to be a mechanosensor 23. Therefore, it is conceivable that alkalinization of the medium imposes a mechanic stress to the yeast cell surface (see below, section "OTHER STRESS SIGNALING PATHWAYS ACTIVATED BY ALKALINE pH STRESS"). In the case of Mid1, this stress would increase its ability to mediate calcium uptake, whereas depolarization associated to alkalinization of the cell surface would activate Cch1. This concerted effect would promote the fast calcium entry and calcineurin activation characteristics of the response to high pH stress.

*In budding yeast *the calcium-activated Ser/Thr protein phosphatase calcineurin is a heterodimer, composed of one of two redundant catalytic subunits, encoded by *CNA1* and *CNA2*, plus a sole regulatory subunit, encoded by the gene *CNB1* 24. The importance of calcineurin in high pH tolerance in *S. cerevisiae* was illustrated by the early report that *cnb1* mutants or wild-type cells treated with the calcineurin inhibitor FK-506 were sensitive to alkalinization of the medium 25,26 and is highlighted by the observation that more than 40% of the deletion mutants found to be sensitive to chemical inhibition of calcineurin by the drug FK-506 were also sensitive to alkaline pH 18. The calcineurin pathway appears necessary for adaptation to alkaline environments not only in *S. cerevisiae*, but also in pathogenic fungi, such as *Candida albicans* 27 or *Cryptococcus neoformans 28.*

A major target of calcineurin is the zinc-finger transcription factor Crz1 (also known as Tcn1 and Hal8). Calcineurin dephosphorylates Crz1 29,30,31, which then enters the nucleus and binds to specific sequences (5′-GNGGC(G/T)CA-3'), known as *C*alcineurin *D*ependent *R*esponse *E*lements (CDRE), present in the promoters of calcineurin-responsive genes, such as the Na^+^-ATPase *ENA1* 29,31,32. Yoshimoto and coworkers showed that among the genes whose expression is affected upon addition of calcium or sodium cations to the medium, around 160 genes are largely dependent on calcineurin and, in most cases, also on the presence of Crz1 33. The calcineurin/Crz1 tandem is conserved in fungi and other lower eukaryotes (see 34 for a recent review).

**Figure 1:**
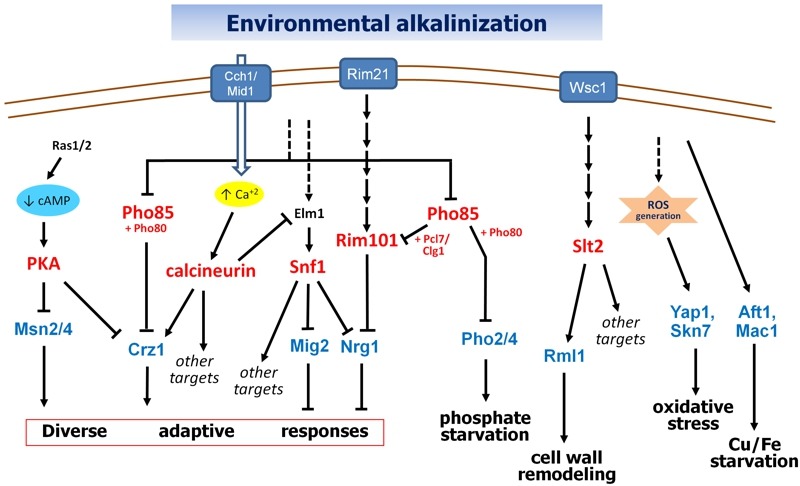
FIGURE 1: Known signaling pathways in alkaline pH response. Discontinuous lines denote still uncharacterized links. See text for details.

The importance of calcium signaling in the transcriptional response to high pH stress was pointed out by Serrano and coworkers 35, who showed that the calcineurin/Crz1 pathway significantly contributed to the rapid induction of *ENA1* and *PHO89 *upon alkalinization of the medium. Subsequent work demonstrated that calcineurin is responsible for the induction of a significant subset of alkali-responsive genes 18. Activation of calcineurin upon alkali stress can be explained by a very fast (seconds) entry of calcium from the extracellular medium into the cell through the Cch1-Mid1 high-affinity calcium channel. The transient rise in intracellular calcium activates calcineurin and promotes entry of Crz1 into the nucleus and binding to its targets in a matter of a few minutes after pH increase 36. ChIP-Seq data generated in our laboratory (S. Petrezsélyová and J. Ariño, unpublished results) revealed an enrichment of Crz1 at around 190 gene promoters in response to alkaline pH stress, of which 24 were previously identified as Crz1-dependent upon alkaline conditions 18,36. Crz1 has been identified as a direct target for several protein kinases. Phosphorylation of Crz1 by Hrr25 (a homolog of human casein kinase 1δ) or the Pho80/Pho85 kinase stimulates nuclear exclusion of the transcription factor 37,38, whereas phosphorylation by PKA would inhibit its nuclear import 39. Since the activity of PKA and Pho85 is affected by alkalinization of the medium (see below), regulation of Crz1 appears at the crossroad of different signaling pathways (Figure 1).

In contrast with the remarkable sensitivity of calcineurin-deficient strains to high pH, *crz1 *mutants have been reported to be insensitive or only marginally affected by alkaline pH 29. This suggests that the Crz1-mediated transcriptional response has a relatively minor role for survival at high pH, and that additional targets for calcineurin, with a relevant role in high pH adaptation, must exist. Possible examples of these targets could be the tail-anchored ER membrane proteins Hph1 and Hph2 (renamed as Frt1 and Frt2). *hph1* *hph2* cells show impaired growth under alkaline pH, salt stress, and cell wall-damaging agents. Hph1, but not Hph2, interacts with and is a substrate of calcineurin. Thus, calcineurin positively modulates Hph1 in a Crz1-independent fashion 40. More recent work has revealed that Hph1 and Hph2 are novel components of the Sec63/Sec62 posttranslational translocation complex that aid in vacuolar proton ATPase biogenesis 41. This function could be at the basis of the alkali-sensitive phenotype of the *hph1* *hph2 strain, since *cells with impaired V-ATPase function cannot properly acidify the vacuole and, consequently, fail to grow at alkaline pH. In this regard, it is known that the electrochemical gradient between the cytosol and vacuolar lumen created by the V-ATPase is required for transport of diverse metabolites and ions, among them, calcium. As mentioned above, exposure to high pH triggers a potent and almost immediate entry of extracellular calcium. It has been shown that cells lacking V-ATPase activity (*vma*^-^ mutants) lose viability after a brief exposure to elevated extracellular Ca^2+ ^and that under these circumstances, the V-ATPase-driven Vcx1 H^+^/Ca^2+^ antiporter is the main activity restoring cytosolic Ca^2+ ^concentrations 42. Therefore, high pH-induced rise in cytosolic calcium is likely to be particularly detrimental in *vma*^-^ cells and proper sequestration of calcium could be one of the roles of the vacuole upon high pH stress.

### THE PARTICIPATION OF THE Rim101 PATHWAY IN THE ALKALINE RESPONSE

Although initially identified in *S. cerevisiae* as a positive regulator of meiotic gene expression and sporulation 43, the role of Rim101 in mediating alkaline pH adaptation and signaling in budding yeast became evident by comparison with the well-established pH-responsive pathway of its homolog in *Aspergillus nidulans*, PacC 16,44. In fact, recent evidence suggest that, in budding yeast cells forming a colony, alkali signaling between cells likely promotes the expansion of the region of sporulation in a way that depends on Rim101-mediated signaling 45.

Rim101 is a C_2_H_2 _zinc-finger transcription factor that binds to TGCCAAG sequences in the promoters of responsive genes, and its activation pathway resembles in many aspects that of *A. nidulans* PacC. The system includes a plasma-membrane located putative sensor system, composed by the 7 transmembrane proteins Rim21 (equivalent to *A. nidulans* PalH) and Dfg16, the 3 transmembrane Rim9 (PalI) protein, and the α-arrestin Rim8 (PalF). It has been proposed that a depolarization of the plasma membrane, caused either by external alkalinization or other mechanisms, can activate the signal transduction in a Rim21-dependent manner (whereas Dfg16 and Rim9 would only have auxiliary functions) 46. The mechanism also involves the activation of Rim101 by alkaline pH-stimulated C-terminal proteolytic cleavage, and it was proposed that the proteolytic complex containing the Rim13 protease is formed and activated by an endocytic mechanism on a ESCRT-III endosomal membrane complex (see 47 for a review). However, evidence has been accumulating that PacC and Rim101 signaling occurs not at the late endosome via endocytosis, but at the plasma membrane by recruitment of downstream components of the pathway 48,49,50, (see 51 for a recent review), and that in *S. cerevisiae* such recruitment involves Rsp5-mediated ubiquinitation events of unknown components other than Rim8 48.

In contrast to PacC, which acts directly as transcriptional activator of "alkaline" genes and as a repressor of "acid" genes, in *S. cerevisiae* Rim101 exerts its role as a repressor, thus controlling the expression of two other transcription factors, *NRG1* and *SMP1 *52. A *rim101* mutant shows high pH sensitivity and a decreased expression of the Na^+^-ATPase encoding gene *ENA1*, and both phenotypes are abolished by deletion of *NRG1*, encoding a transcriptional repressor, thus suggesting that the role of Rim101 in alkaline stress (and salt stress) would be mediated by its effect on the expression of *NRG1 *52. Since Nrg1 function is known to be inhibited by the Snf1 protein kinase 53,54, and Snf1 is activated by alkaline stress 55,56, Nrg1 appears to be at the convergence of two high pH stress-activated pathways (see Figure 1 and below, section "THE CONTRIBUTION OF THE Snf1 PATHWAY TO THE RESPONSE TO ALKALINE pH").

It is worth noting that Rim101 is also involved in weak acid stress resistance 57, which suggests that Rim101 may retain some functionality at acidic conditions. This raises the question of whether cells might share overlapping mechanisms of resistance to both kinds of stresses. We have assembled a list of 292 alkaline pH sensitive mutations from existing literature 7,8,22 and crossed it with the set 629 mutants sensitive to acetic described in Mira *et al.* 58, obtaining a subset of 95 deletion strains sensitive to both stresses. The overlap, a 2.5-fold excess from what could be expected by selecting genes randomly, was enriched only in genes related to vacuolar acidification, which could be anticipated since this process has been proposed to support recovery of cytosolic pH upon treatment of the cells with weak acids, and mutants in the V-ATPase are highly sensitive to alkaline pH. On the other hand, analysis of the set of genes induced by both alkaline stress and acetic acid exposure yielded 33 genes. Gene Ontology analysis revealed an enrichment only in genes related to the broad category "response to stress" (*p*-value 2.20E-04). Therefore, it seems that the overlap in the response mechanisms to both kinds of stresses is very limited. This is not surprising, as it has been demonstrated that the transcription factor Haa1 is a major determinant in the transcriptional response to acetic acid stress 59, whereas this factor seems to play little role in the transcriptional response to high pH stress, nor its mutation renders cells sensitive to this condition.

Among other functions, the cyclin-dependent kinase Pho85 is involved in the transcriptional response to phosphate starvation and alkaline stress (see below). A strain lacking Pho85 is moderately sensitive to high pH 8, and mutation of *rim101* suppresses the growth defect of *pho85* cells under this condition 60. Interestingly, Rim101-dependent genes, such as *NRG1*, are strongly repressed in a *pho85 *strain, and these cells accumulate the repressor in the nucleus. The same authors showed that phosphorylation of Rim101 plays an important role in determining its cellular location and that Pho85 could phosphorylate Rim101 *in vitro*, although they could not obtain evidence for *in vivo* phosphorylation 60. Therefore, although the mechanism is still unclear, the evidence suggests that Pho85 exerts a negative effect on Rim101 function that could be relieved upon inhibition of Pho85 by alkalinization of the medium.

Alkaline pH is likely to cause cell wall damage and results in fast activation of the Slt2 pathway (see below, section "OTHER STRESS SIGNALING PATHWAYS ACTIVATED BY ALKALINE pH STRESS"). It has been reported that the Rim101 pathway contributes to cell wall remodeling in parallel with the protein kinase C (Pkc1)-Slt2 pathway and that it becomes essential in the absence of the Slt2 MAP kinase 61. Interestingly, this role seems to be conserved for the PacC/Rim101 pathway in pathogenic fungi such as *C. albicans* or *C. neoformans* 62,63,64.

### THE PKA PATHWAY IS TRANSIENTLY INHIBITED BY ALKALINIZATION OF THE ENVIRONMENT

The PKA pathway has great relevance in nutrient sensing and stress responses 65. It has been reported that sudden acidification of the cytosol results in transitory increases in cAMP levels, thus activating the PKA pathway 66,67. More recent work has demonstrated that mutations that activate the PKA pathway, such as *ira1 ira2* or *bcy1 *cause sensitivity to alkaline pH, whereas deactivation of the pathway increases high pH stress tolerance 68. In the same work it is shown that alkalinization causes a transient decrease in cAMP and the rapid (2-5 min) nuclear localization of Msn2, a transcription factor that, together with Mns4, contributes to PKA-dependent regulation of the expression of STRE-controlled genes 69,70. Indeed, genome-wide transcriptomic analysis revealed that Msn2/Msn4 contributes to the short-term (10 min) up-regulation of nearly 50% of the genes induced by alkaline pH stress, whereas at longer times (30 min) the relevance of Msn2/Msn4 in the response substantially decreases 68. Therefore, inhibition of the PKA pathway by alkaline pH represents a substantial component of the adaptive response to alkalinization of the environment.

Casado and coworkers also reported that only mutations in the Ras1/Ras2 branch, upstream adenylate cyclase (Cyr1) and PKA, affected alkaline pH tolerance, whereas mutations in diverse genes involved in the Gpr1/Gpa2 glucose sensor system do not produce phenotypic effects 68. These observations, and the fact that the uptake of glucose is not impaired by alkalinization of the medium 56, could indicate that the impact of alkaline pH on the PKA pathway occurs in response to alteration of intracellular glucose metabolism and not to defects in the transport process and/or extracellular glucose detection. Recently, Broggi and coworkers 71 showed that high pH stress affects localization of Ras2, which becomes transiently excluded from the nucleus and translocates to the plasma membrane, and that the treatment triggers a fast increase in the Ras2-GTP/total Ras2 ratio. The authors postulate that alkalinization down-regulates the cAMP/PKA pathway acting on element(s) downstream Ras and that the increase of Ras2-GTP might be explained by a decrease of the reported feedback inhibition operated by PKA on Ras2 72.

The activation state of the PKA pathway is likely to influence on other signaling pathways affected by high pH stress. For instance, as mentioned above (section "THE CALCINEURIN PATHWAY AND THE ALKALINE pH STRESS RESPONSE"), PKA can phosphorylate Crz1, thus opposing the action of calcineurin 39. Therefore, inhibition of PKA may result in further enhancement of calcineurin/Crz1-mediated responses. In addition, it was reported that, upon calcium treatment, Crz1 may exert a destabilization effect on Msn2 levels 73. Since alkalinization of the medium triggers a rapid burst of intracellular calcium, it is conceivable that high-pH induced entry of Crz1 in the nucleus may serve, in addition to activate calcineurin/Crz1-responsive promoters, as a negative-feedback system to avoid persistent activation of Msn2/4-responsive genes, thus explaining the transient Msn2/4-dependent transcriptional effect observed by Casado and coworkers (see above).

### THE CONTRIBUTION OF THE Snf1 PATHWAY TO THE RESPONSE TO ALKALINE pH

Exposure to severe alkaline stress (pH 8.0-8.2) results in rapid induction (10-15 minutes) of a substantial number of genes involved in hexose transport and carbohydrate metabolism, such as high-affinity glucose transporters and TCA cycle enzymes-encoding genes 18,22 that are normally subjected to glucose repression. This set of genes largely overlaps with that of induced when cells are shifted to low glucose-containing medium or reach the diauxic shift 74, pointing that alkaline stress may perturb glucose utilization. This notion was confirmed by the analysis of TCA cycle flux changes after switching cells to pH 7.5, which revealed a substantial increase in TCA cycle activity and respiration rate 75.

The protein kinase Snf1 is the yeast homolog of the mammalian AMP-activated protein kinase and it is required for adaptation of yeast cells to glucose limitation and for utilization of alternative, less preferred carbon sources. In response to glucose limitation, Snf1 is phosphorylated at Thr210 by three upstream Snf1 kinases (Sak1, Elm1 and Tos3), leading to Snf1 activation (see 76,77 for reviews).

In addition to glucose scarcity, Snf1 has been shown to be activated by other unfavorable conditions, such as salt or oxidative stress. Snf1 is also related to alkaline pH stress. Cells lacking Snf1 are markedly sensitive to alkaline pH 55,78,79,80,81 and exposure to high pH results in increased Snf1 phosphorylation and activation 55,82. Increasing the amount of glucose in the medium fully abolishes the *snf1 *alkali-sensitive phenotype and attenuates high pH-induced Snf1 phosphorylation at Thr210 56, suggesting that the role of Snf1 on glucose metabolism appears to be important for its function on high pH tolerance. The same authors demonstrated that most likely the positive input on Snf1 elicited by alkaline pH is generated by the Elm1 kinase, since lack of Elm1 markedly increases alkali sensitivity and this phenotype is abolished by high amounts of glucose in the medium. The *sak1 tos3 *double mutant showed no alkali-sensitive phenotype, but sensitivity of the *tos3 elm1 sak1 *triple mutant was even more pronounced than that of *snf1 *cells and poorly rescued by glucose supplementation, suggesting an important function of these kinases in high pH tolerance besides its regulatory role on the Snf1 kinase. The specific signal(s) activating these kinases in response to high pH stress have not been explored.

**Figure 2:**
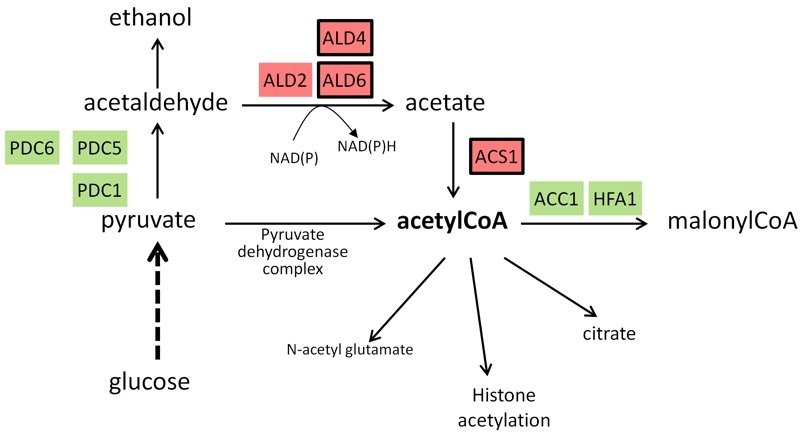
FIGURE 2: Schematic depiction of reactions involved in generation and use of Acetyl CoA. Genes induced in response to alkaline pH stress are in red background whereas those repressed are in green background. Expression profiles have been collected from published transcriptomic data 14,18,22,56,68,97. Genes whose expression has been found to be dependent of Snf1 according to 56 are framed.

Transcriptomic analysis of *snf1* mutants revealed that about 75% of the genes induced in the short-term by high pH are also induced by glucose scarcity, pointing again the relevance of alkaline pH stress on carbohydrate metabolism. It was found that Snf1 mediates, in full or in part, the activation of a significant subset (38%) of short-term alkali-induced genes, including those encoding high-affinity hexose transporters and phosphorylating enzymes, and glycogen (but not trehalose) metabolism 56. As depicted in Figure 2, the expression profile of diverse genes metabolically linked to the generation of Acetyl CoA shows a tendency to favor the synthesis of this compound and to avoid its transformation in malonyl CoA. In this regard it is worth noting that Snf1 phosphorylates and inhibits the acetyl-CoA carboxylase Acc1, the enzyme that catalyzes carboxylation of cytosolic acetyl-CoA to generate malonyl-CoA. Recently, Zhang and coworkers reported 83 that decreased expression of *ACC1* results in increased acetyl-CoA levels and suppresses diverse phenotypes, including alkaline pH sensitivity, in the *snf1* mutant. In addition, the low levels of acetyl-CoA of *snf1* mutant cells and the alkaline pH sensitivity can be suppressed by deletion of *MIG1*, which is a repressor of the acetyl-CoA synthetase, encoded by the *ACS1 *gene. Therefore, the authors conclude that some of the phenotypes and transcriptional defects of *snf1* cells are due to their low acetyl-CoA level, which results in global histone hypoacetylation 83. These findings suggest that a possible role for Snf1 in response to alkaline stress could be to promote the expression of *ACS1* and to limit the activity of *ACC1*, thus ensuring appropriate acetyl-CoA availability. This notion fits with transcriptional data showing that *ACS1* is induced by high pH stress 22,56,68 and that this effect is partially dependent on the presence of Snf1 56, whereas the same treatment decreases *ACC1* mRNA levels 14,18 (see Figure 2).

Interestingly, it has been reported that many genes that are both repressed by glucose and rapidly induced by alkaline stress are also under the direct positive regulation of the calcineurin/Crz1 pathway 36. In fact, activation of calcineurin (i.e. by addition of calcium to the medium) was sufficient to allow growth of a *snf1* mutant under limiting glucose availability. Therefore, alkalinization of the medium would generate two independent signals, activation of calcineurin (through the influx of external calcium) and Snf1 (by activation of the Elm1 kinase) that, in the short-term, would represent independent but combined strategies to cope with alkaline pH stress. Very recently it has been reported that calcineurin can dephosphorylate and inactivate the Elm1 kinase during alkaline stress 84, perhaps providing a negative feedback mechanism to avoid excessive activation of the system. This feedback mechanism might explain the transient nature of Snf1 phosphorylation upon high pH stress 82.

### OTHER STRESS SIGNALING PATHWAYS ACTIVATED BY ALKALINE pH STRESS

The cell wall integrity (CWI) signaling pathway is essential for growth and required for tolerance to cell wall damaging agents. Briefly, the pathway is composed of several, partially overlapping membrane sensors (Wsc1-4, Mid2, and Mtl1) which in response to cell wall stress interact with the GDP/GTP exchange factors Rom2 or Rom1 leading to activation of the small GTPase Rho1. GTP-Rho1 interacts with and activates the Pkc1, the only protein kinase C in budding yeast. In turn, Pkc1 activates a typical MAP kinase module, consisting of Bck1, the apparently redundant pair of MAPK kinases Mkk1 and Mkk2, and, finally, the Slt2 MAPK (see 85 for a review). Activation of Slt2 results in the phosphorylation of several cytosolic and nuclear targets, such as the transcription factors Rlm1 and the SCB-binding factor (Swi4 and Swi6). Strains defective for key components of this pathway display a lytic phenotype when exposed to cell wall damaging agents (i.e. Calcofluor white or Congo Red) or increased temperature*, *but this phenotype can be suppressed by addition of sorbitol to the medium.

As far as we know there is no available information about the chemical changes in cell-wall composition of yeast cells subjected to alkaline stress. However, it has been shown that the sensitivity to zymolyase (composed mainly of β-1,3-glucanase activity) of cells grown at pH 6.0 is higher than those grown at pH 4.0, while the relative proportion of β-1,6-glucan decreases around 40% 86. In any case, a number of evidences suggest that high pH stress causes cell wall damage. The link between alkaline pH and cell wall stress was established years ago, on the basis of two independent genome-wide screens that identified *bck1* and *slt2* mutants as sensitive to high pH 7,8. In fact, Gene Ontology analysis of a list of 289 mutants sensitive to high pH that we have compiled from the literature 7,8,22 shows a substantial enrichment in cells related to cell wall organization or biogenesis (7.2E-6). In addition, there is a remarkable overlap between the set of genes induced in response to severe alkaline stress 18 and those found induced after exposure to cell wall damaging agents, such as zymolyase or caspofungin, or in mutants for certain genes encoding cell wall components 87,88. Serrano and coworkers reported that Slt2 resulted rapidly (5 min) and transiently activated after alkalinization of the medium, and this response was depended on the integrity of the MAP kinase module 22. These authors also found that either lack of Wsc1 or specific mutations in this sensor impairing sensing or transduction of the signal, rendered cells alkali-sensitive and reduced the activation of Slt2 by high pH, whereas constitutive activation of Slt2 by the *bck1-20 *allele increased pH tolerance in the *wsc1 *mutant. These results support the notion that Wsc1 might act as sensor for high pH stress and that activation of the Slt2 MAP kinase module is required for normal high pH tolerance. The fact that Wsc1 has been identified as a cell surface mechanosensor 23 further suggests that alkaline stress causes alterations at the cell surface.

It has become evident in the last years that, in addition to Slt2, other signaling pathways could be relevant in the response to cell wall stress, such as the one mediated by Rim101 61 or by the Hog1 MAP kinase 89,90. However, whereas alkaline pH certainly activates the Rim101 pathway, this is not the case for the Hog1 pathway 22,91. In contrast, activation of the Snf1 pathway by high pH stress might also contribute to preserve cell wall integrity, as it has recently shown that a *snf1* mutant is hypersensitive to different compounds or treatments that cause cell wall stress, and that phosphorylation of Snf1 at Thr210 in response to treatment with the glucan synthase inhibitor caspofungin is important for the function of Snf1 in cell wall integrity 92. Genetic evidence suggests that the role of Snf1 would be independent of the Slt2 cell wall integrity pathway.

Yeast cells exposed to high pH stress accumulate in relatively short time reactive oxygen species, suggesting they are also confronted with a certain degree of oxidative stress 18. This explains the observation that cells lacking *SOD1* or *SOD2*, encoding superoxide dismutases, are sensitive to high pH 8 and that a substantial number of genes that are induced in response to oxidative stress, such as *PRX1*, *GPX2, GRX1*, *TRR1* or *TRX2,* are also induced by alkalinization of the medium 18,35. This transcriptional response involves the transcription factors Yap1 and, probably in lesser extent, Skn7. A role for Snf1 in signaling oxidative stress has been proposed, since oxidative stress provokes a modest activation of the kinase. The observation that the *snf1* mutant is not particularly sensitive to hydrogen peroxide 55,93, casts some doubts about its role in anti-oxidative defense. However, more recent work has proved that yeast cells require Snf1 (in an Elm1-dependent manner) for normal growth in the presence of diethyl maleate, diamide, or the pro-oxidant selenite, all of which cause alterations in glutathione redox homeostasis by increasing the levels of oxidized glutathione 94. Therefore, the possibility that Snf1 could participate in the response to oxidative stress caused by alkalinization of the medium still remains open to speculation. Very recently, a protective role for Rim101 in cells treated with diverse oxidants, including diamide and selenite, has been reported by Pérez-Sampietro and coworkers 95. This effect, similarly to the alkaline pH response, would require Rim8, the ESCRT-I/II/III complexes and the Rim13 protease involved in proteolytic activation of Rim101, as well as Nrg1, the transcriptional repressor downstream Rim101. These authors postulate that the protective effect of Rim101 in cells confronted with selenite is based on the regulatory role of Rim101 in vacuolar homeostasis and its requirement for normal vacuolar acidification. Therefore, the function of Rim101 in adaptation to alkaline stress might also include a role in confronting the associated oxidative stress.

### ALKALINE STRESS AND NUTRIENT HOMEOSTASIS

In yeast cells uptake of most nutrients rely on active transport processes that are based in symport with protons and thus the majority of nutrients transporters are dependent on the proton gradient. Among others, these include some sugar transporters, all amino acids transporters, organic acid transporters, anion transporters (such as phosphate and sulfate), some monovalent (i.e. potassium) or divalent (i.e. magnesium, zinc) cations. Therefore, it can be anticipated that alkalinization of the medium would have a notorious impact on nutrient homeostasis. These effects were highlighted by early works describing the genome-wide transcriptional response to high pH stress.

As mentioned above, high pH stress provokes a rapid induction of genes that are normally subjected to glucose repression. These includes high-affinity glucose transporters, as well as glycolytic and TCA cycle enzymes-encoding genes 18,22,36, generating a pattern that is reminiscent of that observed when cells are shifted to low glucose-containing medium or they reach the diauxic shift 74. However, although increasing the amount of glucose in the medium from the standard 2% up to 6% significantly improves growth at pH 8.0 (but not at pH 5.5) and normalizes high pH tolerance of the hypersensitive *snf1* strain 56, alkalinization of the medium does not impairs glucose uptake 56, so it is likely that in this case the signal is generated inside the cell possibly due to the perturbation of glucose utilization. It is well known that the PKA, Snf1 and TOR pathways have key roles in nutrient sensing 96. However, whereas the first two pathways play important roles in adaptation to alkalinization of the medium, the role of the TOR pathway is questionable, since mutants in most genes involved in the pathway, with the exception of the pleiotropic *ure2* mutant, do not show a high pH-sensitive phenotype 8.

Shifting cells to high pH also results in an important alteration in copper and iron homeostasis. This is likely caused by the decrease in solubility of these metals in alkaline conditions and is reflected in that:

i) alkaline stress gives rise to increased mRNAs levels for genes encoding iron (*FET3, FRE1, FET4*), siderophores (*ARN1*, *ARN2*, *ARN3*, *ARN4*, *FIT2*) or copper (*CTR1*, *CTR3*) transporters 35,97,

ii) mutant strains lacking *CTR1* (encoding the main high-affinity copper transporter) or *AFT1* (the transcription factor required for activation of target genes in response to changes in iron availability), as well as other mutants related to copper and iron homeostasis, such as *ccs1*, *ccc2* or *fet3,* are also alkali-sensitive 8, and

iii) only two genes,* FET4 *and *CTR1,* were able to increase high pH tolerance when overexpressed in a wild type strain 8. Further work demonstrated that the beneficial effect of overexpression of *CTR1 *was largely due to the improvement in iron uptake, highlighting the tight connection between copper and iron metabolisms 98. Addition to the medium of micromolar concentrations of these metals drastically improves growth at high pH 8.

Collectively, these results emphasize the fact that copper and iron availability are important limiting factors for growth under alkaline pH conditions. It should be noted that diverse genes involved in vacuolar acidification have been shown to be necessary for growth under limiting iron conditions 99. Since an equivalent situation is triggered by high pH, this might point to a possible role of vacuole acidification in normal alkaline stress tolerance. In addition, several enzymes involved in antioxidant defenses are metalloenzymes requiring iron or copper. Thus, catalases (such as yeast Ctt1) possess iron-containing heme groups 100, whereas superoxide dismutases (such as Sod1) are copper/zinc containing enzymes. Therefore, it cannot be excluded that high pH is capable of negatively affecting cellular antioxidant defenses.

Expression of *CTR1* and *FRE1* is regulated by the Mac1 transcription factor, and *FET3* is under the control of Aft1. It is worth noting that Pho85 is required for proper expression of *MAC1 *and *AFT1* 101. Given the various links between Pho85 and alkaline pH signaling (see above and below), the sensitivity of a *pho85 *strain under alkaline conditions 8,9,60 might have diverse origins. In addition, expression of *FET4* has been reported to be, in part, Rim101-dependent 52.

Alkaline stress results in the activation of many genes involved in phosphate (Pi) acquisition or metabolism, such as *PHO84*, *PHO89*, *PHO11,* *PHO12, VTC1*, or* VTC4, *which are also up-regulated during Pi starvation, suggesting that alkalinization of the medium mimics a situation of Pi scarcity. This is reasonable if it is considered that low-affinity Pi transport is based on H^+^/Pi symport 102,103,104. It has been postulated that increasing the pH of the medium decreases the ionic (monovalent) form of phosphate, which is the proposed uptake form for a yeast cell 105. However, this effect could be also explained by the fact that the major high-affinity Pi transporter, Pho84, is also a H^+^/Pi transporter, so that at high pH its uptake capacity is far from optimal. In that case, the capacity of Pho89, able to work at alkaline pH, would be clearly lower than that of low-affinity and Pho84 transporters 106.

**Figure 3:**
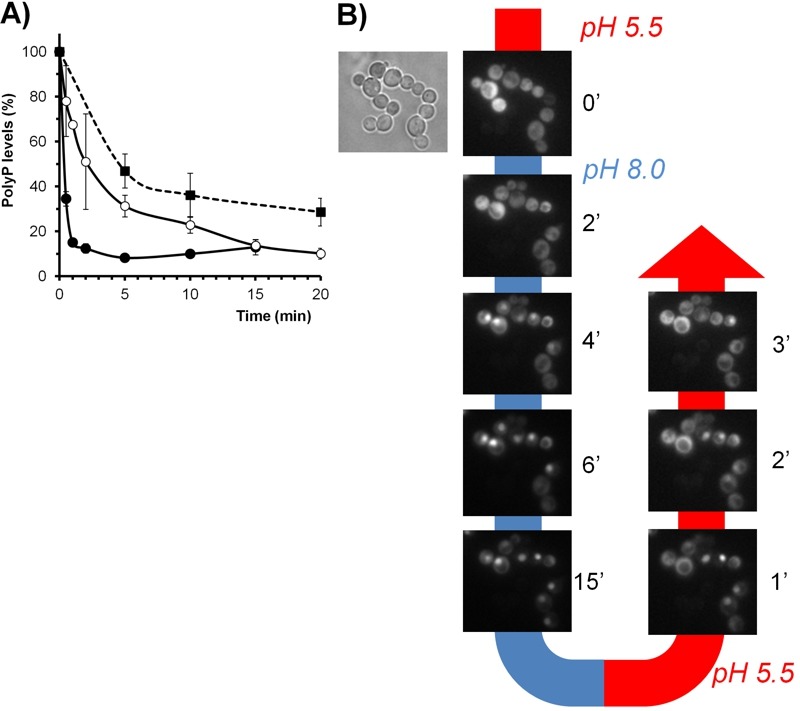
FIGURE 3: Effects of alkaline pH stress on phosphate metabolism. **(A)** Depletion of intracellular PolyP stocks upon alkalinization of the medium. Strains BY4741 (wild type, closed circles) and its isogenic derivative DCS30 (*ppn1* *ppx1, *open circles) were grown up to OD_600_ 0.6 and subjected to alkaline stress (pH 8.0) as in 82. For comparison, in parallel experiments the wild type strain was subjected to phosphate limitation (0.1 mM Pi) as in 126. Samples of the culture were taken at the indicated times and polyP content determined as in 126. Data is expressed as percentage relative to the levels just prior initiation of the stress and are mean ± SEM from 3 independent experiments. **(B)** Reversible cytosolic-nuclear transfer of Pho4 upon modification of the extracellular pH. Wild type strain DBY746 was transformed with plasmid Pho84-GFP, carrying a C-terminally GFP-tagged version of *PHO4*, and grown until OD_660_ 0.6 in Verduyn medium 127. Aliquots were injected into a CellASIC^®^ ONIX Y04C-02 microfluidics chamber and the pH of the medium changed with time as indicated in the figure. Images were collected with a Nikon Eclipse TE2000-E microscope using a FITC filter and a Hamamatsu ORCA-ER camera.

The response of *PHO84 *(a high-affinity H^+^/Pi co-transporter) and *PHO12* (a secretable phosphatase) to alkaline pH was shown to be fully dependent of Pho2/Pho4 35, the transcription factors that mediate the transcriptional responses to phosphate starvation, and of the cyclin-dependent kinase inhibitor Pho81, which was considered an intracellular phosphate sensor 107. Alkalinization of the medium was shown to trigger degradation of the polyPs stock 108, and our results (Figure 3A) indicate that this is a very fast process. In fact, the decrease in polyP reserves induced by alkaline stress was faster than that observed upon shifting cells to phosphate limitation. This decrease was only partially blocked in cells lacking *PPN1* and *PPX1 *(Figure 3A), the genes encoding the presumed major polyP phosphatase activities 109, thus supporting the notion that additional polyP phosphatase activities should exist. In any case, the yeast vacuole is commonly considered the major storage site for polyP, which is regarded as a cellular phosphate buffer that can be mobilized during phosphate limitation 110. Since alkaline stress subjects the cell to a situation similar to that of phosphate starvation, and it is known that most V-ATPase mutants fail to accumulate significant levels of polyP 111, it is conceivable that maintenance of normal levels of polyP could be one of the contributions of normal vacuolar acidification to high pH stress tolerance.

Alkalinization of the medium also provoked the fast entry of Pho4 into the nucleus, which was rapidly reversed upon acidification of the medium (Figure 3B). Induction of *PHO84* and *PHO12 *after high pH shock is a relatively late event (45-120 min) 35,97, and the kinetics of accumulation of Pho84 upon high pH stress are very similar to that observed upon phosphate starvation 82. All these results indicate that, in this case, the signaling pathway activated by alkaline pH is the same that is triggered by phosphate starvation. Consistently, mutation of *PHO2*, *PHO4* or *PHO81 *conferred an alkali-sensitive phenotype, which was particularly dramatic for the *pho84* *pho89* mutant 8,9. In contrast, induction of *PHO89* is an early (10-15 min) and transient event. *PHO89* encodes a Na^+^/Pi co-transporter that, in contrary to Pho84, is mainly active at alkaline pH 112. In addition to regulation by Pho2/Pho4, our earlier work demonstrated that expression of *PHO89* upon alkaline stress is strongly regulated by calcineurin/Crz1 35. The complex response of *PHO89* and its physiological implications will be discussed in the next section.

### INTEGRATION OF DIFFERENT SIGNALING PATHWAYS IN HIGH pH-RESPONSIVE GENE PROMOTERS

The great diversity of signaling pathways with an impact on gene expression that are modulated by alkaline pH stress likely reflects the very broad impact that environmental alkalinization has on the biology of the yeast cell. Therefore, it is not surprising that several high-pH activated pathways may impinge on a given gene promoter, thus finely tuning the response. An example is represented by genes such as *HXT2*, *HXT7*, *ALD4* or *ADH3,* which are normally induced in response to the scarcity of glucose through activation of Snf1 and, upon alkalinization of the medium, becoming also sensitive to activation mediated by calcineurin/Crz1. This dual mechanism has physiological significance since, as mentioned above in the section "THE CONTRIBUTION OF THE Snf1 PATHWAY TO THE RESPONSE TO ALKALINE pH", activation of calcineurin by external calcium was shown to override the growth defect of a *snf1* mutant in limiting glucose conditions 36. Similarly, a recent publication reported that under alkaline conditions the inhibition of Pho85 and the subsequent activation of Pho4, Crz1 and Rim101 contributed to the downregulation of *CLN2*, probably leading to an alteration of the cell cycle progression 113.

**Figure 4:**
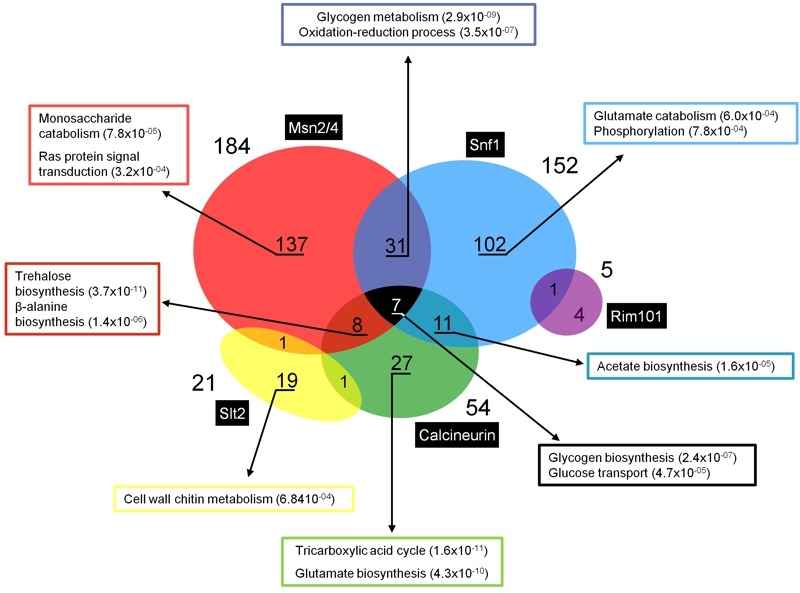
FIGURE 4: Genes regulated by multiple signaling pathways in response to alkaline pH stress. Expression profiles have been collected from published transcriptomic data 18,22,36,56,68,97. Gene Ontology analyses were based on FuncAssociate 2.0 128 and YeastMine 129. Numbers in parentheses denote the *p*-value for a given category.

We have analyzed existing transcriptomic data to evaluate, at the genome-wide level, how frequent the incidence of two or more regulatory pathways is in the control of the expression of a given gene in response to high pH stress. The dataset encompasses comparisons between the profile of the wild type strain with those of *msn2 msn4, snf1, cnb1/crz1, slt2 *or* rim101 *mutants. The entire dataset shows nearly 1000 genes that are induced in at least one of the experiments at a given time-point (it must be noted that individual datasets were obtained in different experimental conditions, i.e. pH of the medium ranging from 7.6 to 8.2). As illustrated in Figure 4, induction of 349 genes depends on the presence of at least one of the factors described above, and the most prevalent regulatory components appears to be the PKA pathway, through the Msn2/Msn4 transcription factors, and the Snf1 pathway. Sixty genes (17%) appear to be multiregulated. Among them, 38 genes appear to be induced by the contribution of the Snf1 and Msn2/Msn4 pathways, and this set is enriched in genes related to glycogen metabolism and oxido-reduction processes. Eighteen genes are under the control of Snf1 and calcineurin, with significant presence of acetate biosynthetic genes, whereas 15 genes are regulated by calcineurin and Msn2/Msn4, among them several related to trehalose and β-alanine biosynthesis (Figure 4). Remarkably, up to 7 genes, all of them related to carbohydrate metabolism (*GSY2*, *PGM2*, *MDH2*, *HXT7*, *GSY1*, *HXK1*, and *HXT5*) are regulated by 3 different pathways: Snf1, Msn2/Msn4 and calcineurin. Finally, the genes regulated by Slt2 and Rim101 are less interconnected with the other pathways, perhaps due to the specificity of the target genes controlled by these pathways.

Regulatory inputs are particularly complex in the promoter of the *ENA1* Na^+^-ATPase gene, a key determinant for saline stress tolerance (see 91 for a review). Under alkaline pH stress, the *ENA1* promoter integrates signals from at least 3 major pathways: calcineurin (via Crz1 at two CDRE) 32, Snf1 (via both Mig1/Mig2 and Nrg1), and the Rim101 pathway, via Nrg1 32,81. Although the *ENA1* promoter contains several putative GATA sequences 114, which would suggest it is under the control of the TOR pathway, they are likely irrelevant in response to high pH stress. Similarly, under pH stress, *ENA1* does not seem to be regulated by the Msn2/Msn4 transcription factors 35, although the inhibition of the PKA pathway might led to hypophosphorylation of Crz1, thus reducing the chances for inactivation of the transcription factor (see above, section "THE PKA PATHWAY IS TRANSIENTLY INHIBITED BY ALKALINIZATION OF THE ENVIRONMENT"). Remarkably, a very recent report highlighted that, in response to high pH stress, *ENA1 *and* PHO89 *share the same regulatory network 82 and that both proteins accumulate roughly at the same time at the plasma membrane. The fact that *PHO89* promoter is responsive to calcineurin, Snf1 and Rim101 explains its fast activation upon alkaline stress. In addition, it allows establishing a physiological link between Pho89 and Ena1 functions and justifies the puzzling observation that Ena1 is induced not only by salt stress (very reasonable for a Na^+^-ATPase) but also by alkaline pH even in the absence of salt stress. At high pH, Pho89 becomes an important contributor to Pi uptake 106. However, this has a trade-off: the co-transport of Na^+ ^cations. Therefore, rapid induction of *ENA1* would be a safety measure not only to avoid accumulation of Na^+ ^up to toxic levels, but also because increasing intracellular Na^+^ would eliminate the extracellular/intracellular cation gradient necessary to sustain uptake of Pi through Pho89. Consequently, even under mild alkaline conditions, a *pho84* *ena1* mutant phenocopies the extreme growth defect of a *pho84* *pho89* strain, a mutant devoid of high-affinity Pi transport 82. The widespread presence of Pho89-like transporters among eukaryotes and the requirement for Ena1 to grow at alkaline pH in the absence of salt stress, observed in diverse fungi and in bryophytes (see 115 and references therein) suggest that the regulatory coupling between Pho89 and Ena1 transporters might be evolutionarily conserved.

### CONCLUSIONS

Shifting yeast cells from the standard acidic environment to a moderately alkaline medium transiently alters the mRNA level of hundreds of genes. Whereas this review essentially focuses on the signaling pathways impinging on the transcriptional regulation of high pH responsive genes, it must be kept in mind that the mRNA level of a given gene is the result of two opposing forces: the activity of the promoter and the stability of the generated mRNA. The relevance of changes in mRNA stability in response to stress conditions has been recognized in the last few years and investigated in some cases, such as osmotic, oxidative or heat-shock stresses 116,117. In collaboration with the laboratory of J.E. Pérez-Ortín (University of Valencia), our group has recently characterized with the aid of the Genomic Run-On technology (GRO) the effect of environmental alkalinization on transcription rate and mRNA stability 118. It was found that alkalinization of the media to pH 8.0 causes an overall decrease in transcription rate and a fast destabilization of mRNAs, followed by a more prolonged stabilization phase. In some cases, as in genes responsive to oxidative stress, the increment on mRNA levels occur without a relevant increase in the transcription rate, and is probably attributable to mRNA stabilization of these genes. In comparison to other forms of stress previously analyzed such as oxidative, osmotic and heat-shock stresses, alkaline stress response is atypical because of the limited contribution of transcription rate to the increase in mRNA levels. In contrast, the decrease in the amount of mRNAs is contributed by a decline in both the transcription rate and mRNA stability. Since there is growing evidence that mRNA decay is also a subject for regulation, it seems necessary to focus future work in this direction to gain full understanding of the adaptive response to high pH stress.

Expanding our knowledge of the adaptive mechanisms of yeasts to alkalinization may have a relevant impact on technological or health-related applications. We have mentioned above that supplementing the medium with minimal amounts of copper and iron cations markedly increases tolerance to high pH, an effect that is also achieved by overexpression of the *CTR1* and *FET4* metal transporters 8. More recently, it has been shown that expression of *Kluyveromyces lactis* *FAD2* and *FAD3* genes in *S. cerevisiae* leads to accumulation of linoleic and alpha-linolenic acids and results in increased alkaline pH tolerance, as well as to Li^+^ and Na^+^ cations 119. This effect was related to the activation of the Rim101 pathway. Improving tolerance to high pH in yeast by a combination of genetic manipulation and conditioning of the growth medium could be useful for industrial applications requiring growth of yeast at pH higher than normal. On the other hand, adaptation to the wide range of extracellular pH found in the different hosts’ environments is required for pathogenic fungi for survival.

Interestingly, evidence has been accumulating in support of a role of several signaling pathways described in this review in drug resistance. A relationship between tolerance to antifungal drugs and calcineurin was described in *C. albicans*, *A. fumigatus* and *C. neoformans, *and the combination of drugs such as azoles or caspofungin with calcineurin inhibitors leads to an increased susceptibility to the drugs 120,121,122. The Pac/Rim pathway was also found to be involved in tolerance to azoles (see 123 and references therein), and similar results were observed in *C. neoformans *124. The relevance of pH signaling in fungal pathogenesis in humans can be probably extended to fungal plant pathogens. Thus, in the rice pathogen *Magnaporthe oryzae,* deletion of *the RIM101/PacC homolog PACC, *caused a progressive loss in growth rate from pH 5 to pH 8, a loss in conidia production at pH 8 *in vitro*, and a partial loss in virulence towards barley and rice, suggesting that PacC plays an important role during interaction between the *M. oryzae* and its host plants 125.
